# Supplementary material for: The Systems Biology Research Tool: evolvable open-source software
Source: BMC Syst Biol. 2008 Jun 29;2:55. doi: 10.1186/1752-0509-2-55 (PMC2446383; doi:10.1186/1752-0509-2-55)
Supplement: Additional file 1 — SBRT Archive. An archive of the current version of the Systems Biology Research Tool. [file 1752-0509-2-55-S1.zip › sbrt-1.4.0/doc/developers_guide/api/sbrt/shell/mng/package-use.html]

Uses of Package sbrt.shell.mng


|  |  |  |
| --- | --- | --- |
| **Overview** \| **Package** \| Class \| **Use** \| **Tree** \| **Deprecated** \| **Index** \| **Help** | |  |
| PREV   NEXT | **FRAMES**    **NO FRAMES**     **All Classes** |  |


---


## **Uses of Package sbrt.shell.mng**

| Packages that use sbrt.shell.mng | |
| --- | --- |
| **sbrt** | Provides the class used to run the Systems Biology Research Tool. |
| **sbrt.shell** | Provides classes used by shell-level software. |
| **sbrt.shell.io** | Provides classes and interfaces for reading and writing files. |
| **sbrt.shell.mng** | Provides classes and interfaces for creating process managers. |
| **sbrt.shell.mng.algebra** | Provides classes for creating algebra process managers. |
| **sbrt.shell.mng.comb** | Provides classes for creating combinatorics process managers. |
| **sbrt.shell.mng.fba** | Provides classes and interfaces for creating Flux Balance Analysis process managers. |
| **sbrt.shell.mng.geom** | Provides classes and for creating geometry process managers. |
| **sbrt.shell.mng.graph\_theory** | Provides classes and for creating graph theoretical process managers. |
| **sbrt.shell.mng.stat** | Provides classes and interfaces for creating statistical process managers. |
| **sbrt.shell.mng.utilities** | Provides classes and interfaces for creating mathematical process managers. |

Classes in sbrt.shell.mng used by sbrt

****ManagerInput****
  
          This interface is used to represent the input to
`KernelProcessManager` objects.

Classes in sbrt.shell.mng used by sbrt.shell

****ManagerInput****
  
          This interface is used to represent the input to
`KernelProcessManager` objects.

****ProcessManager****
  
          This interface is used to represent process managers.

Classes in sbrt.shell.mng used by sbrt.shell.io

****ManagerInput****
  
          This interface is used to represent the input to
`KernelProcessManager` objects.

Classes in sbrt.shell.mng used by sbrt.shell.mng

****ManagerInput****
  
          This interface is used to represent the input to
`KernelProcessManager` objects.

****ProcessManager****
  
          This interface is used to represent process managers.

Classes in sbrt.shell.mng used by sbrt.shell.mng.algebra

****AbstractProcessManager****
  
          This class is a skeleton implemenation of `KernelProcessManager`.

****KernelProcessManager****
  
          This interface is used to represent process managers.

****ManagerInput****
  
          This interface is used to represent the input to
`KernelProcessManager` objects.

****ProcessManager****
  
          This interface is used to represent process managers.

****ShellProcessManager****
  
          This interface is used to represent shell process managers.

Classes in sbrt.shell.mng used by sbrt.shell.mng.comb

****AbstractProcessManager****
  
          This class is a skeleton implemenation of `KernelProcessManager`.

****KernelProcessManager****
  
          This interface is used to represent process managers.

****ManagerInput****
  
          This interface is used to represent the input to
`KernelProcessManager` objects.

****ProcessManager****
  
          This interface is used to represent process managers.

Classes in sbrt.shell.mng used by sbrt.shell.mng.fba

****AbstractProcessManager****
  
          This class is a skeleton implemenation of `KernelProcessManager`.

****KernelProcessManager****
  
          This interface is used to represent process managers.

****ManagerInput****
  
          This interface is used to represent the input to
`KernelProcessManager` objects.

****ProcessManager****
  
          This interface is used to represent process managers.

****ShellProcessManager****
  
          This interface is used to represent shell process managers.

Classes in sbrt.shell.mng used by sbrt.shell.mng.geom

****AbstractProcessManager****
  
          This class is a skeleton implemenation of `KernelProcessManager`.

****KernelProcessManager****
  
          This interface is used to represent process managers.

****ManagerInput****
  
          This interface is used to represent the input to
`KernelProcessManager` objects.

****ProcessManager****
  
          This interface is used to represent process managers.

Classes in sbrt.shell.mng used by sbrt.shell.mng.graph\_theory

****AbstractProcessManager****
  
          This class is a skeleton implemenation of `KernelProcessManager`.

****KernelProcessManager****
  
          This interface is used to represent process managers.

****ManagerInput****
  
          This interface is used to represent the input to
`KernelProcessManager` objects.

****ProcessManager****
  
          This interface is used to represent process managers.

****ShellProcessManager****
  
          This interface is used to represent shell process managers.

Classes in sbrt.shell.mng used by sbrt.shell.mng.stat

****AbstractProcessManager****
  
          This class is a skeleton implemenation of `KernelProcessManager`.

****KernelProcessManager****
  
          This interface is used to represent process managers.

****ManagerInput****
  
          This interface is used to represent the input to
`KernelProcessManager` objects.

****ProcessManager****
  
          This interface is used to represent process managers.

Classes in sbrt.shell.mng used by sbrt.shell.mng.utilities

****AbstractProcessManager****
  
          This class is a skeleton implemenation of `KernelProcessManager`.

****KernelProcessManager****
  
          This interface is used to represent process managers.

****ManagerInput****
  
          This interface is used to represent the input to
`KernelProcessManager` objects.

****ProcessManager****
  
          This interface is used to represent process managers.

---


|  |  |  |
| --- | --- | --- |
| **Overview** \| **Package** \| Class \| **Use** \| **Tree** \| **Deprecated** \| **Index** \| **Help** | |  |
| PREV   NEXT | **FRAMES**    **NO FRAMES**     **All Classes** |  |


---
